# Immunohistochemical Expression of Serine and Arginine-Rich Splicing Factor 1 (SRSF1) in Fluoro-Edenite-Induced Malignant Mesothelioma: A Preliminary Study

**DOI:** 10.3390/ijerph18126249

**Published:** 2021-06-09

**Authors:** Giuseppe Broggi, Giuseppe Angelico, Veronica Filetti, Caterina Ledda, Claudia Lombardo, Ermanno Vitale, Venerando Rapisarda, Carla Loreto, Rosario Caltabiano

**Affiliations:** 1 Department of Medical and Surgical Sciences and Advanced Technologies, F. Ingrassia, Anatomic Pathology, University of Catania, 95123 Catania, Italy; giuseppe.broggi@gmail.com (G.B.); rosario.caltabiano@unict.it (R.C.); 2 Unità di Gineco-patologia e Patologia Mammaria, Dipartimento Scienze della Salute della Donna, del Bambino e di Sanità Pubblica, Fondazione Policlinico Universitario A. Gemelli IRCCS, 00168 Roma, Italy; giuangel86@hotmail.it; 3 Human Anatomy and Histology, Department of Biomedical and Biotechnology Sciences, University of Catania, 95123 Catania, Italy; verofiletti@gmail.com (V.F.); claudialombardodoc@gmail.com (C.L.); carla.loreto@unict.it (C.L.); 4 Occupational Medicine, Department of Clinical and Experimental Medicine, University of Catania, 95123 Catania, Italy; cledda@unict.it (C.L.); ermannovitale@gmail.com (E.V.)

**Keywords:** malignant mesothelioma, fluoro-edenite, SRSF1, prognostic factor

## Abstract

The Serine and Arginine-Rich Splicing Factor 1 (SRSF1) has a proto-oncogenic function, being associated with angiogenesis and frequently overexpressed in many human malignant neoplasms. Its immunohistochemical expression has never been investigated in malignant pleural mesothelioma (MPM). We evaluated SRSF1 immunoexpression and its possible relation to angiogenesis in a selected cohort of 10 fluoro-edenite(FE)-induced MPM cases. Methods: Immunohistochemical analyses with an anti-SRSF1 antibody were performed. We interpreted the cases as positive if tumor cell nuclei were stained; a semi-quantitative analysis of the cases was performed by evaluating the intensity of staining and the percentage of tumor positive cells. A microvessel density (MVD) count was also performed. Results: High and low immunoexpressions of SRSF1 were seen in six and four MPMs, respectively. A trend of shorter overall survival was found in FE-induced MPM patients with SRSF1 overexpression. In addition, a significant association between high-MVD and high SRSF1 immunoexpression (*p* = 0.0476) was found. Conclusions: SRSF1 appears to be involved in MPM pathogenesis and its immunoexpression may represent a prognostic biomarker capable of identifying subgroups of patients with different prognosis. However, given the preliminary nature of the present study, further investigations on larger series, and additional in vitro studies, are required to validate our findings.

## 1. Introduction

Malignant pleural mesothelioma (MPM) is a malignant tumor originating from the mesothelial layer of the pleura and traditionally related to the exposure to asbestos fibers [1]. Despite the strong association with occupational or residential exposure to asbestos fibers, other asbestos-like fibers, including erionite and fluoro-edenite (FE) fibers, have been demonstrated as alternative pathogenetic agents capable of promoting MPM [2,3,4]. In detail, epidemiological studies performed between 1988 and 1997 demonstrated high incidence and mortality rates of MPM in Biancavilla, a small town near Mt. Etna in Sicily (Italy), linked to environmental and occupational exposure to FE fibers [4,5,6,7,8,9,10,11,12,13,14,15,16]. These latter fibers were isolated in the lava rocks excavated from a local stone quarry and used for about 50 years for building purposes [4,5,6]. Subsequent studies demonstrated their morphological and size similarities with tremolite amphibolic asbestos fibers [4,5,6]; therefore, FE fibers have been declared carcinogenic by the International Agency for Research on Cancer (IARC; Lyon, France) [7]. MPM carries a poor outcome, given its low rates of response to treatments and it is often diagnosed at an advanced stage; therefore, the median survival is approximately 6 to 12 months [4,5,6,7,8,9,10,11,12,13,14,15,16].

Different diagnostic immunohistochemical markers are currently available for MPM, such as calretinin, CK5/6, podoplanin, mesothelin, osteopontin, hyaluronic acid, fibulin-3 and vascular endothelial growth factor [3]. In actual fact, tumor stage, histological subtype, sex and age at diagnosis represent the most important prognostic parameters in MPM patients [17]. Recently, some authors have also emphasized the prognostic and predictive role of the water channel protein aquaporin-1 (AQP1) in MPM patients [17,18]. However, reliable prognostic and predictive biomarkers for improving MPM patient management, are yet to be discovered.

The Serine and Arginine-Rich Splicing Factor 1 (SRSF1) is a member of the SR protein family involved in constitutive and alternative pre-mRNA splicing [19]; additional functions include mRNA transcription regulation, stability and nuclear export, nonsense-mediated mRNA decay and translation [19]. SRSF1 has also been identified as a proto-oncogene, associated with angiogenesis and frequently overexpressed in many solid tumors, including breast, brain, colon, liver and lung tumors [20,21,22,23,24]. However, its expression and functions in MPM have never been investigated.

The aim of the present study was to evaluate SRSF1 immunoexpression and its possible relation to neoangiogenesis in MPM cases related to FE exposure.

## 2. Materials and Methods

### 2.1. Ethics Statement and Sample Collection

Although the present research complied with the Helsinki Declaration, the non-interventional retrospective nature of our study did not require any informed consent by the local research ethics committee.

Clinico-pathological data from 49 surgically treated, MPM patients between 1996 and 2014 were retrospectively collected. All patients were residents in the town of Biancavilla and showed evidence of environmental exposure to FE. For ten of these patients, adequate thoracoscopic bioptic tissue and follow-up data were available. The following inclusion criteria for evaluating the adequacy of the histologic specimens were adopted: (i) tumor paraffin blocks had to contain enough neoplastic tissue to cut additional slides for immunohistochemistry; (ii) they had to contain representative tumor tissue; (iii) extensive necrosis did not have to be present to not alter the immunoreactivity of the neoplastic cells.

### 2.2. Laboratory Tests and Evaluation of SRSF1 Immunohistochemistry

Formalin-fixed and paraffin-embedded specimens were cut to 4–5 μm, mounted on sialinate-coated slides (Dako, Glostrup, Denmark), stored at room temperature and stained with hematoxylin and eosin. Pathological diagnosis of MPM was rendered according to WHO criteria. In addition, for each case, immunohistochemical investigation was carried out using antibodies anti-SRSF1 (sc-33652; working dilution 1:50; Santa Cruz Biotechnology, Dallas, TX, USA) and anti-CD31 (JC70A; working dilution 1:40; DAKO, Glostrup, Denmark).

**Figure 1:**
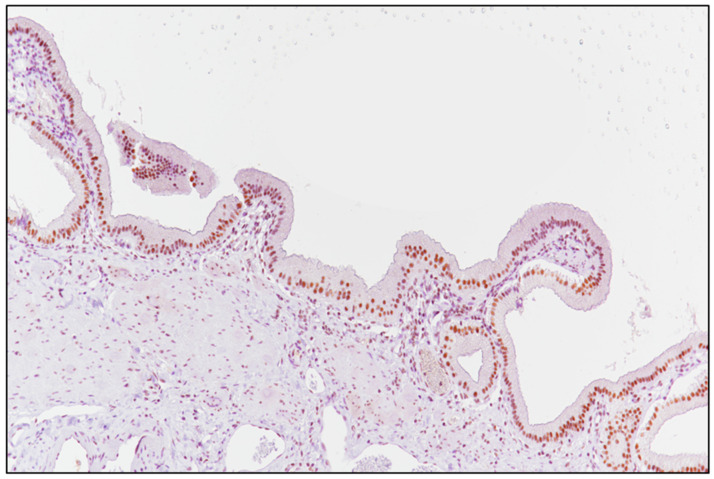
Unaffected gallbladder mucosa served as a positive control for SRSF1 (immunoperoxidase staining; original magnification 150×).

The detection of brown chromogen within tumor nuclei was considered as positive SRSF1 immunostaining; unaffected gallbladder tissue was adopted as a positive control (Figure 1), while negative control slides were obtained by incubating them with phosphate-buffered saline (PBS) instead of the primary antibody. A semi-quantitative analysis of the cases stained with SRSF1 was performed, as previously described [25,26,27]: briefly, the immunoreactivity score (IRS) was obtained by multiplying the intensity of staining (IS) and the percentage of positive cells (extent score; ES): if the IRS was ≤6, the SRSF1 expression was considered to be “low” (L-IRS), while an IRS > 6 was considered to be “high” expression (H-IRS).

### 2.3. Blood Vascular Microvessel Density (MVD)

The evaluation of microvessel density (MVD) was performed by three pathologists (G.B., G.A. and R.C.), as previously described [28,29]. Vascular hotspots were highlighted on tissue sections by immunohistochemistry using an anti-CD31 immunohistochemical antibody (JC70A; working dilution 1:40; DAKO, Glostrup, Denmark) at 4× and 10× magnifications. MVD represented the total amount of vessels/mm^2^ (conversion factor: 1 mm^2^ = 4 high power fields- HPFs-). Areas with ≥50 of viable tumor tissue were included in the count; the following exclusion factors were considered: extensive necrosis, hemorrhage and desmoplasia. Endothelial cells stained with CD31 and each lumen for long branched vessels were counted. Finally, small clusters of at least 2 stained endothelial cells within the same vessel were counted as a single one. Cases showing a value more than the median of immunoreactive vascular structures were considered as evidence of high MVD, while cases with MDV values above the median were considered as low MVD.

### 2.4. Statistical Analysis

We compared the rate of high and low levels of SRSF1 expression in MM. The Hazard Ratio (HR) was calculated using the Mantel–Haenszel test. Cancer-specific survival analysis and the comparison of the survival curves were performed using the Kaplan–Meier method and the Mantel–Cox log-rank test, respectively. To evaluate the correlation between clinical–pathological and immunohistochemical data, the Spearman correlation was performed. *p*-values less than 0.05 (*p* < 0.05) were considered as statistically significant. Statistical analysis was performed using GraphPad Prism version 7 (GraphPad Software, Inc., La Jolla, CA, USA).

## 3. Results

### 3.1. Clinico-Pathological Features of the Patients Included in the Study

The cohort of patients affected by FE-related MPM included six men and four women with a mean age of 68.4 years (age range: 50–93 years). Based on the World Health Organization (WHO) criteria, six cases were histopathologically diagnosed as epithelioid MPMs, one case as sarcomatoid MPM, and three cases as biphasic MPMs [30]. Among the biphasic MPMs, two cases exhibited a mild predominance of the sarcomatoid component (60% sarcomatoid vs. 40% epithelioid), while the remaining one showed an almost “pure” spindle cell morphology with only scattered glandular elements. Table 1 summarizes the clinico-pathological and immunohistochemical features of the cases from our cohort.

### 3.2. SRSF1 Immunohistochemical Expression and Its Correlation with Prognosis

**Figure 2:**
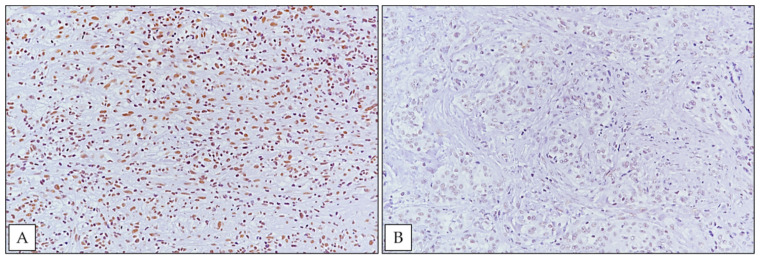
(**A**) Strong nuclear immunohistochemical expression of SRSF1 in a case of biphasic MPM (immunoperoxidase staining; original magnification 200×); (**B**) weak SRSF1 immunoexpression in an epithelioid MPM case from our cohort (immunoperoxidase staining; original magnification 200×).

**Figure 3:**
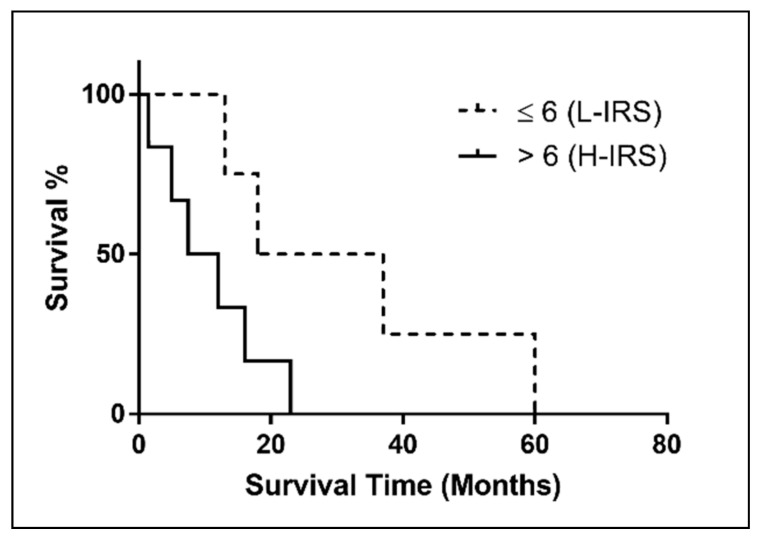
Kaplan–Meier survival curve of SRSF1 expression (IRS) in FE-induced MM patients. (*p* = 0.0563).

SRSF1 was detected with a high immunoexpression (Figure 2A) in 60% (*n* = 6) of MPM FE-induced cases, while 40% (*n* = 4) of cases showed low immunostaining (Figure 2B). Considering the median overall survival (OS) between high and low SRSF1 expression, there was no significant association between SRSF1 expression and increased OS (*p* = 0.0563), and the hazard ratio (HR) was 0.2461 with a 95% confidence interval (CI) (0.05833 to 1.038) (Figure 3). No significant relationship between SRSF1 expression and other clinico-pathological variables (age, sex and MM pathological subtype) was observed. Moreover, a trend of shorter OS was found in FE-induced MPM patients with SRSF1 overexpression. By contrast, the better prognoses were depicted in the 40% of the cases that exhibited a low immunoexpression of SRSF1. In detail, a correlation between SRSF1 overexpression and decreased survival times was found (mean OS time of only 9.75 months for patients with high expression vs. mean OS of 27.5 months for patients with low SRSF1 expression) (Figure 3).

### 3.3. SRSF1 Immunoexpression Was Positively Associated with MVD Levels

**Figure 4:**
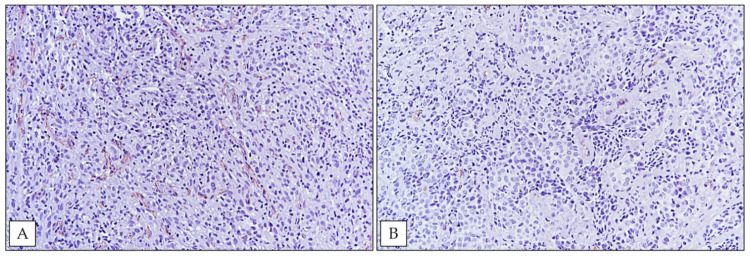
(**A**) High MVD in a biphasic MPM (immunoperoxidase staining; original magnification 200×); (**B**) an epithelioid MPM case exhibiting low MVD (immunoperoxidase staining; original magnification 200×).

Taking into consideration a cut-off < or >91 (median MVD value, Table 1), five cases showed high-MVD (Figure 4A) and the remaining five cases were considered low-MVD (Figure 4B). Interestingly, Fisher exact test, showed a significant association between high-MVD and high SRSF1 immunoexpression (*p* = 0.0476) (Table 2). In detail, five of six cases with high SRSF1 immunoexpression exhibited high MVD values; on the other hand, all cases showing low SRSF1 immunohistochemical expression exhibited this at the same time as low MVD values.

## 4. Discussion

The splicing factor SRSF1 has recently been demonstrated as a proto-oncogene, frequently overexpressed in different solid tumors. SRSF1 is also involved in regulating the activity of proto-oncogenes, tumor suppressors and apoptotic regulator genes [19,20,21,22,23,24].

To date, only few studies have investigated SRSF1 protein status in human tissues [19,20,21,22,23,24]. Moreover, its immunohistochemical expression in MPM, as well as its possible relation to clinicopathological parameters, has never been investigated. In the present study, we provide the first evidence of SRSF1 expression in MPM.

In detail, according to our SRSF1-IRS, the high immunohistochemical expression of SRSF1 was detected in 6/10 (60%) MPM cases, while a low expression was observed in the remaining 4/10 (40%) cases.

Regarding the relations between SRSF1 immunohistochemistry and clinico-pathological factors, a shorter OS was observed in MPM patients with SRSF1 overexpression. In contrast, longer OSs were observed in MPM cases that showed a low immunohistochemical expression of SRSF1. In detail, mean OSs of 9.75 and 27.5 months were, respectively, observed in patients with high and low SRSF1 expression (Figure 3). No other significant relations between SRSF1 expression and other clinico-pathological variables, including age, sex and pathological subtype, were found.

These findings are in line with previous reports concerning SRSF1 expression in other solid tumors, including gliomas and lung cancer, in which its overexpression has been linked to a higher malignancy grade and poorer survival [19,20,21,22,23,24].

In this regard, previous in vitro studies, have demonstrated that high levels of SRSF1 indicate a more invasive phenotype caused by the hyperactivation of the AKT and ERK signaling pathways [19,20,21,22,23,24]. Overexpression of SRSF1 has also been linked to the activation of epithelial to mesenchymal transition (EMT) which induces a more aggressive phenotype, as well as increased resistance to standard chemotherapeutic agents [19,20,21,22,23,24].

In addition, some authors [29,31,32,33] previously demonstrated on human glioblastoma tissue samples that SRSF1 also acted as a pro-angiogenic factor, being part of a molecular axis, mediated by circSMARCA5—a circular RNA that regulated cell migration and angiogenesis through the binding of SRSF1—and involved in the splicing of the Vascular Endothelial Growth Factor A. (VEGF-A). The splicing process of VEGF-A pre-mRNA may alternatively generate both a pro-angiogenic and an anti-angiogenic isoform [29,31,32]; the above-mentioned authors [29,31,32,33] hypothesized that the upregulation of SRSF1 led to an angiogenic stimulation on GBM tissue, through the switch of the proangiogenic/antiangiogenic ratio of VEGF-A.

Given these findings, we performed an MVD evaluation on the FE-induced MPM tissue samples of our cohort to better understand the potential proangiogenic role of SRSF1 in this tumor; notably, our results strongly indicated a possible relationship between SRSF1 expression and neoangiogenesis in MPM. In fact, SRSF1 high and low expressions in our series were related to high and low MVD values, respectively; these results were statistically significant (*p* = 0.0476) (Table 2).

In conclusion, our findings indicate that SRSF1 is involved in MPM pathogenesis and suggest that its immunohistochemical expression may represent a prognostic biomarker capable of identifying low expressor patients with a better prognosis and high expressor patients with a more aggressive phenotype. Further studies on larger series are, therefore, needed to validate and expand our actual comprehension of splicing protein functions in MPM patients; in particular, the possibility of confirming the stimulatory role of SRSF1 on neoangiogenesis on in vitro MPM cell line models represents one of the most interesting future perspectives of this study.

## Figures and Tables

**Table 1:** Clinico-pathological and immunohistochemical features of the MPM cases.

Case	Age (Years)	Gender	Pathological Subtype	Survival Time (Months)	SRSF1 IS	SRSF1 ES	SRSF1 IRS	MVD (*n*/mm^2^)
**1**	69	M	Epithelioid	1.5	3	3	9	132/mm^2^
**2**	50	M	Biphasic (20% Epithelioid, 80% Sarcomatoid)	16	3	4	12	103/mm^2^
**3**	69	F	Sarcomatoid	5	2	4	8	147/mm^2^
**4**	74	F	Epithelioid	13	2	2	4	84/mm^2^
**5**	85	M	Epithelioid	23	2	4	8	79/mm^2^
**6**	93	F	Biphasic (40% Epithelioid, 60% Sarcomatoid)	7.5	3	3	9	139/mm^2^
**7**	58	F	Epithelioid	18	2	3	6	88/mm^2^
**8**	55	M	Epithelioid	37	3	2	6	64/mm^2^
**9**	75	M	Biphasic (40% Epithelioid, 60% Sarcomatoid)	60	2	3	6	28/mm^2^
**10**	56	M	Epithelioid	12	2	4	8	94/mm^2^

**Table 2:** Distribution of MVD scores according to SRSF1 immunostaining.

MVD	High SRSF1	Low SRSF1
High	5	0
Low	1	4

## Data Availability

Not applicable.
